# Membrane-Bound TNF Induces Protective Immune Responses to *M. bovis* BCG Infection: Regulation of memTNF and TNF Receptors Comparing Two memTNF Molecules

**DOI:** 10.1371/journal.pone.0031469

**Published:** 2012-05-30

**Authors:** Maria L. Olleros, Dominique Vesin, Ruth Bisig, Marie-Laure Santiago-Raber, Sonia Schuepbach-Mallepell, George Kollias, Olivier Gaide, Irene Garcia

**Affiliations:** 1 Department of Pathology and Immunology, Centre Medical Universitaire (CMU), University of Geneva, Geneva, Switzerland; 2 Department of Dermatology-Venereology, Geneva University Hospital, Geneva, Switzerland; 3 Biomedical Sciences Research Center Alexander Fleming, Institute of Immunology, Vari-Athens, Greece; University of Maryland, United States of America

## Abstract

**Background:**

Several activities of the transmembrane form of TNF (memTNF) in immune responses to intracellular bacterial infection have been shown to be different from those exerted by soluble TNF. Evidence is based largely on studies in transgenic mice expressing memTNF, but precise cellular mechanisms are not well defined and the importance of TNF receptor regulation is unknown. In addition, memTNF activities are defined for a particular modification of the extracellular domain of TNF but a direct comparison of different mutant memTNF molecules has not been done *in vivo*.

**Methodology:**

To understand the activities of memTNF we compared two commonly used mouse strains lacking soluble TNF but possessing functional and normally regulated membrane-bound TNF knockin (memTNF KI) for their capacity to generate cell-mediated immune responses and resistance to *M. bovis* BCG infection, and to regulate TNF receptors.

**Principal Findings:**

*M. bovis* BCG infection resulted in similar bacterial loads in one strain of memTNF KI (memTNF^Δ1–9,K11E^) and in wild-type mice, in contrast, the other strain of memTNF KI mice (memTNF^Δ1–12^) showed higher sensitivity to infection with high mortality (75%), greater bacterial load and massive lung pathology. The pattern of cytokines/chemokines, inflammatory cells, pulmonary NF-κB phosphorylation, antigen-dependent IFN-γ response, and splenic iNOS was impaired in *M. bovis* BCG-infected memTNF^Δ1–12^ KI mice. Macrophages expressing TNFR2 were reduced but soluble TNFRs were higher in memTNF^Δ1–12^ KI mice during the infection. *In vitro*, *M. bovis* BCG-induced NF-κB activation and cytokines were also decreased in memTNF^Δ1–12^ KI bone marrow-derived macrophages.

**Conclusion:**

Our data show that two memTNF molecules exerted very different activities upon *M. bovis* BCG infection resulting in protection or not to bacterial infection. These results suggest a regulatory mechanism of memTNF and TNF receptors being critical in the outcome of the infection and highlight the role of cell-bound and soluble TNFR2 in memTNF-mediated anti-microbial mechanisms.

## Introduction

Tumor necrosis factor (TNF) is a major pro-inflammatory cytokine playing an important role in the pathogenesis of chronic inflammatory diseases such as rheumatoid arthritis, Crohn's disease or psoriatic arthritis [1]. Most activities exerted by TNF were originally attributed to the soluble form of TNF (solTNF), a 17-kDa soluble TNF molecule generated by cleavage of its 26-kDa precursor, transmembrane or membrane TNF (memTNF). The generation of uncleavable memTNF by mutations in the region cleaved by the TACE (TNF-α converting enzyme) and subsequent studies on cells, transgenic mice and later in memTNF knockin (KI) mice without soluble TNF and in human cells have shown the importance of activities mediated by the transmembrane form of TNF [2], [3]. However, it is still unclear how memTNF-mediated effects are influenced by several factors including the nature of mutations generated on the memTNF molecule, and regulatory mechanisms involving TNFR and their soluble forms which have not been explored *in vivo*. As an example of the reported complexity, studies on the same memTNF expressed in mice either as a transgene or as a knockin have resulted in opposite effects, as a memTNF (deletion 1–12, memTNF^Δ1–12^) was previously shown to mediate inflammatory reactions in a transgenic mouse model [4], while memTNF^Δ1–12^ KI mice did not show liver inflammation after LPS/D-GalN challenge [5]. We have recently reported that another variant of memTNF (deletion 1–9 and substitution Lys to Glu in position 11, memTNF^Δ1–9,K11E^) in KI mice [6] was not pathogenic for liver inflammation but only soluble TNF was causing hepatitis in LPS/BCG and LPS/D-GalN-induced liver damage [7].

Host protection mechanisms mediated by transmembrane TNF against bacterial and parasitic infections have been studied mainly using memTNF^Δ1–9,K11E^ KI mice. It was reported that memTNF^Δ1–9,K11E^ KI mice were protected from low-dose *Listeria monocytogenes* infection but only partially or not protected from high dose infection [8], [9]. A study of *L. monocytogenes* infection in memTNF^Δ1–12^ KI mice showed partial survival of these mice to low bacterial dose [5]. The memTNF^Δ1–9,K11E^ form was shown to be sufficient to develop partial innate immunity to *Francisella tularensis* live vaccine strain [10]. An important role of memTNF^Δ1–9,K11E^ was observed in the resolution of the inflammatory lesion induced by *Leishmania major* infection [11]. Infection with *M. tuberculosis* of memTNF^Δ1–9,K11E^ KI mice showed protection against acute but not to chronic infection [12], [13]. These studies confirmed our previous results on transgenic mice expressing a different mutant memTNF (Δ-12 to −10, Δ-2 to +1, K11E) expressed as a transgene in mice deficient in both TNF and LTα (transgenic memTNF in TNF/LTα^−/−^) [14], [15], [16]. Only one study has been reported for *M. tuberculosis* infection using memTNF^Δ1–12^ KI mice showing protection to acute but not to chronic infection, and the infection dosage were lower than those used in the studies of memTNF^Δ1–9,K11E^ KI mice [17] (Table 1).

**Table 1:** *M. tuberculosis* and *M. bovis* BCG infection studies performed in memTNF KI mice.

MemTNF KI mouse strain	Mycobacteria	Dose and route of inoculation	Survival	References
MemTNF^Δ1–9,K11E^	*M. tuberculosis*	70–100 CFU by aerosol	Succumbed:130–200 days	[13]
MemTNF^Δ1–9,K11E^	*M. tuberculosis*	100 CFU intranasally	Succumbed:110–200 days	[12]
MemTNF^Δ1–9,K11E^	BCG Pasteur	10^6^ CFU intranasally	100% survival at 120 days	[18]
MemTNF^Δ1–9,K11E^	*M. tuberculosis*	50–100 CFU by aerosol	Succumbed: 150–230 days	[18]
**MemTNF^Δ1–12^**	BCG Pasteur	2×10^6^ intravenously	50% survival at 90 days	[17]
**MemTNF^Δ1–12^**	*M. tuberculosis*	10–30 CFU by aerosol	Succumbed: 50–170 days	[17]
MemTNF^Δ1–9,K11E^	BCG Connaught	10×10^6^ intravenously	93% survival at 100 days	Present study
MemTNF^Δ1–9,K11E^	BCG Pasteur	10×10^6^ intravenously	100% survival at 100 days	Present study
**MemTNF^Δ1–12^**	BCG Connaught	10×10^6^ intravenously	30% survival at 100 days	Present study
**MemTNF^Δ1–12^**	BCG Pasteur	10×intravenously	20% survival at 100 days	Present study

Infection with *M. bovis* BCG in memTNF KI mice has shown differential susceptibilities of these mice. MemTNF^Δ1–9,K11E^ KI mice intranasally infected showed total resistance to *M. bovis* BCG infection, whereas memTNF^Δ1–12^ KI mice intravenously (i.v.) infected were only 50% resistant [17], [18]. The reasons of differential susceptibility of the two memTNF KI mouse strains were not addressed in previous publications. To date, a rigorous comparison of immune responses elicited by intracellular bacterial infection in the two memTNF KI mice has not been performed and the importance of TNF receptors during a bacterial infection is unknown.

To gain insights into the role of memTNF and its interaction with TNF receptors in protection against intracellular bacterial infection, we have compared resistance and cell-mediated immunity to *M. bovis* BCG infection of memTNF^Δ1–9,K11E^ KI and memTNF^Δ1–12^ KI mice, and studied the pattern of cytokine and TNF receptor expression, and NF-κB activation. We used a systemic *M. bovis* BCG infection that allows these studies in highly sensitive mice such as TNF−/− mice in which the pattern of inflammatory lesions have been well characterized. Considering that *M. bovis* BCG is the most widely distributed vaccination with live bacteria, deep investigation on the factors influencing protective immunity is still required.

Our results show important differences between the two memTNF KI mouse strains in both resistance and cell-mediated immune responses to *M. bovis* BCG infection. These differences reveal distinct activities mediated by the interaction of either memTNF^Δ1–9,K11E^ KI or memTNF^Δ1–12^ with TNF receptors. Our data show that cell-bound as well as soluble TNFR2 play a critical role in the outcome of the infection.

## Results

### Mice without solTNF and expressing only memTNF^Δ1–9,K11E^ are more resistant to *M. bovis* BCG infection than mice with memTNF^Δ1–12^

**Figure 1:**
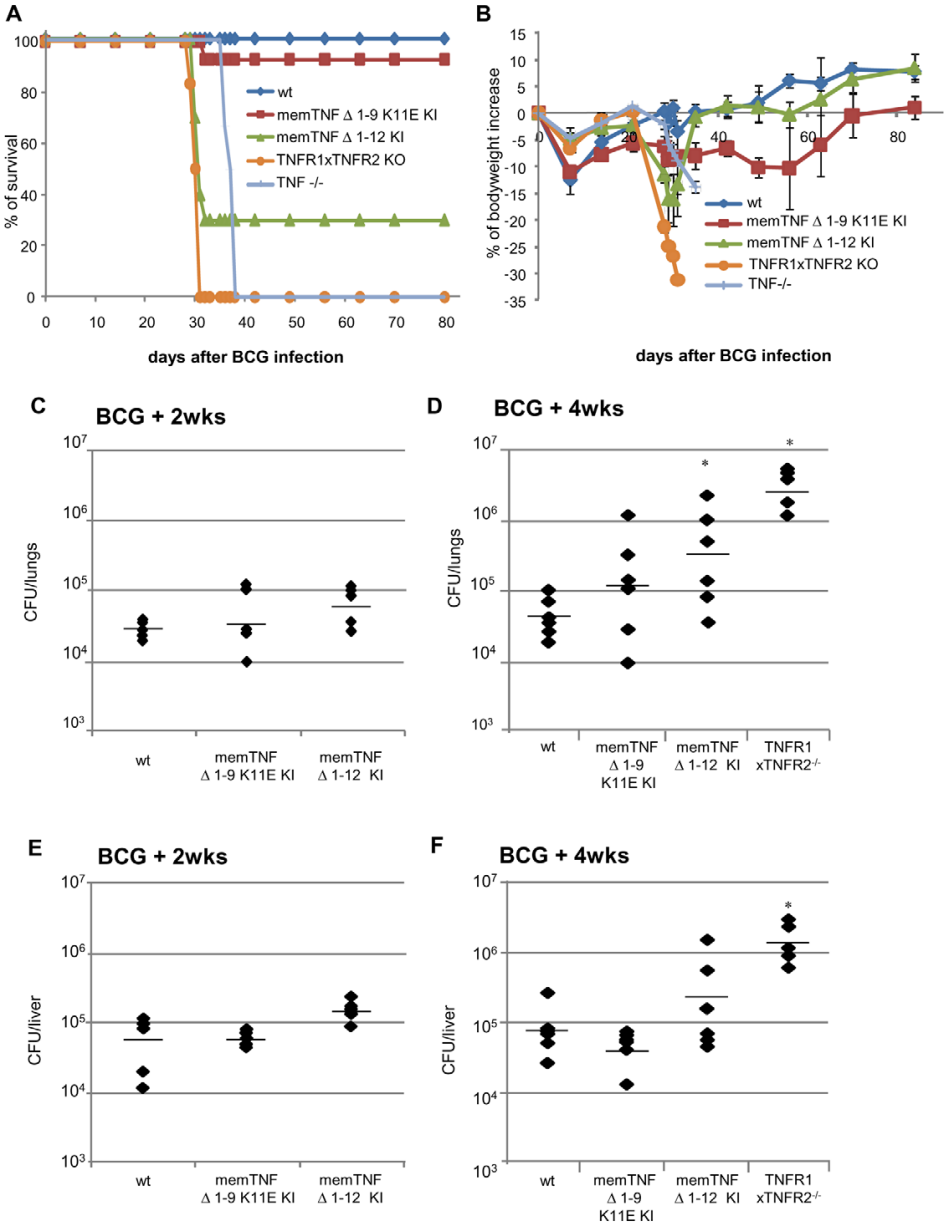
Survival curve, body weight and bacterial loads in lung and liver of mice infected with 10^7^CFU of *M. bovis* BCG. (A) Long-term survival of mice infected with living *M. bovis* BCG Connaught (10^7^). (B) Body weight change after *M. bovis* BCG infection. Wild-type and memTNF^Δ1–9,K11E^ KI, (*n* = 14 mice per group), memTNF^Δ1–12^ (*n* = 10 mice), and TNF−/− mice (*n* = 6 per group). Data from two representative experiments are shown. CFU were determined in lungs (C, D) and liver (E, F) at 2 and 4 weeks after infection with 10^7^ CFU of *M. bovis* BCG. Data are represented as individual values and horizontal bars indicate mean (*n* = 4–6 mice per group). Asterisks indicate statistically significant differences between wild type and indicated group (*, p<0.03).

**Figure 2:**
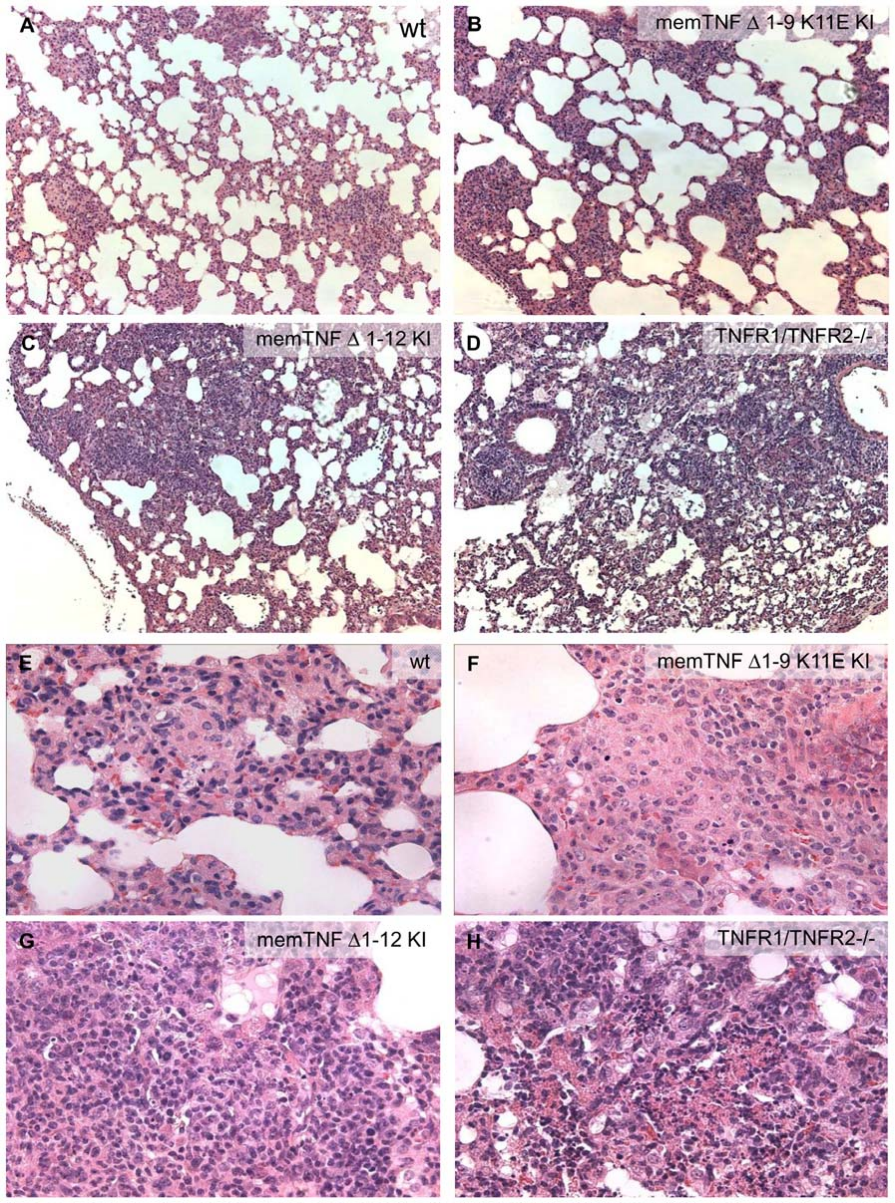
Histopathology of pulmonary lesions 4 weeks after *M. bovis* BCG infection. Wild-type mouse lung (A, E) shows small granulomas containing multinucleated giant cells, memTNF^Δ1–9,K11E^ KI mouse lung (B, F) shows larger granulomas compared with wild-type, also presenting multinucleated giant cells, and memTNF^Δ1–12^ KI (C, G) and TNFR1/TNFR2−/− (D, H) mouse lungs exhibit large lesions containing numerous lymphocytes, neutrophils and monocytes, but lacking multinucleated giant cells. Regions of necrosis are observed in TNFR1/TNFR2−/− lungs. Figure is representative of two experiments (*n* = 6 per group). Magnification A–D 100× and E–H 400×.

To explore the protective activities of the transmembrane form of TNF against *M. bovis* BCG infection, we compared the development of host defense mechanisms against this pathogen in two different memTNF KI mouse strains. We infected wild-type, memTNF^Δ1–9,K11E^ [6] and memTNF^Δ1–12^ [5], double TNFR1/TNFR2−/−, and TNF−/− mice by the i.v. route. Wild-type mice were resistant to *M. bovis* BCG infection; one out of 14 (93% survival) memTNF^Δ1–9,K11E^ KI mice died, whereas 7 out of 10 (30% survival) memTNF^Δ1–12^ KI mice succumbed to the infection with loss of body weight, and all TNFR1/TNFR2 −/− and TNF−/− mice died (Fig. 1A, B). After infection with a different strain of *M. bovis* BCG (Pasteur), we observed total survival of memTNF^Δ1–9,K11E^ KI mice, while 80% of memTNF^Δ1–12^ KI mice succumbed to the infection between days 25 and 45 and exhibited significant loss of body weight (Fig. S1). Infection of memTNF^Δ1–12^ KI mice deficient in TNFR1 or TNFR2 resulted in similar pattern than memTNF^Δ1–12^ KI mice at day 50 after infection (Fig. S1A, B). Therefore, both infections with *M. bovis* BCG strains confirmed the higher susceptibility of memTNF^Δ1–12^ KI mice compared to memTNF^Δ1–9,K11E^ KI and wild-type mice. The enhanced susceptibility of memTNF^Δ1–12^ KI mice versus memTNF^Δ1–9,K11E^ KI mice was confirmed by bacterial load in lungs and liver as shown in Figure 1C–F. Deficiency of either TNFR1 or TNFR2 in memTNF^Δ1–12^ KI mice showed an increased bacterial load in liver and lungs at 4 weeks after *M. bovis* BCG infection as compared to memTNF^Δ1–12^ KI mice, indicating that both receptors could play a role in memTNF^Δ1–12^ KI signalling (Fig. S1C, D). Histopathological examination of lungs at 4 weeks post-infection revealed that memTNF^Δ1–9,K11E^ KI mice formed granulomas of larger size than wild-type mice (average diameter was 197±87 versus 170±30 µm in wild-type) (Fig. 2A, B), and that these granulomas contained multinucleated giant cells as in wild-type pulmonary granulomas (Figure 2E, F). This pathology contrasted with that observed in memTNF^Δ1–12^ KI and TNFR1/TNFR2−/− mice which showed extended pulmonary lesions (average diameter of inflammatory foci was 310±100 µm and 481±120 µm, p<0,01 and p<0,02 vs wild-type, respectively) (Fig. 2C, D) exhibiting abundant lymphocytes and absence of multinucleated giant cells (Fig. 2G, H). In particular, TNFR1/TNFR2−/− mice developed extensive necrotic pulmonary lesions (Fig. 2H).

### Different cytokine, chemokine, inflammatory cell and NF-κB activation during *M. bovis* BCG infection in memTNF^Δ1–9,K11E^ and memTNF^Δ1–12^ KI mice

**Figure 3:**
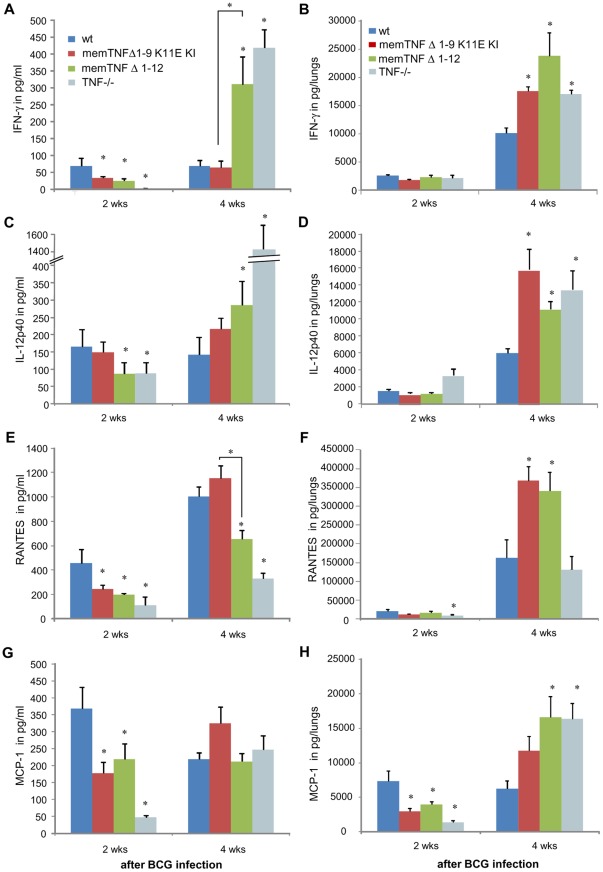
Serum and lung levels of cytokines and chemokines after *M. bovis* BCG infection. IFN-γ levels were assessed in serum (A) and in lungs (B) at 2 and 4 weeks after *M. bovis* BCG infection. Serum (C) and lung (D) amounts of IL-12p40 were measured at 2 and 4 weeks after *M. bovis* BCG infection. Data are mean ± SEM and are representative of two independent experiments (*n* = 6–10 per group). *, p<0.03. RANTES (E, F) and MCP-1 (G, H) amounts were measured in serum (E, G) and in lungs (F, H) at 2 and 4 weeks after *M. bovis* BCG infection. Data are represented as means ± SEM from two independent experiments (*n* = 4–6 per group). *, p<0.04.

To determine whether memTNF^Δ1–9,K11E^ KI mice and memTNF^Δ1–12^ KI mice are able to induce Th-1 type cytokines as well as chemokines upon *M. bovis* BCG infection, the levels of IFN-γ, IL-12p40, RANTES and MCP-1 were evaluated in the serum and lungs and compared to those of wild-type mice. IFN-γ serum levels of memTNF^Δ1–9,K11E^ KI and memTNF^Δ1–12^ KI mice were lower than those in wild-type mice at 2 weeks, but at 4 weeks after *M. bovis* BCG infection, exacerbated expression of IFN-γ was observed only in memTNF^Δ1–12^ KI and TNF−/− mice, whereas memTNF^Δ1–9,K11E^ KI mice exhibited similar level to wild-type mice (Fig. 3A). In the lung, IFN-γ amounts were similar in all groups of mice at 2 weeks and increased in all mutant mice 4 weeks post-infection (Fig. 3B). IL-12p40 serum amounts were comparable in memTNF^Δ1–9,K11E^ KI and wild-type mice but lower in memTNF^Δ1–12^ KI and TNF−/− mice at 2 weeks, and at 4 weeks, they were higher in memTNF^Δ1–12^ KI, and TNF−/− mice (Fig. 3C). IL-12p40 lung content also revealed increased values in all mutant mice at 4 weeks infection compared to wild-type mice (Fig. 3D).

**Figure 4:**
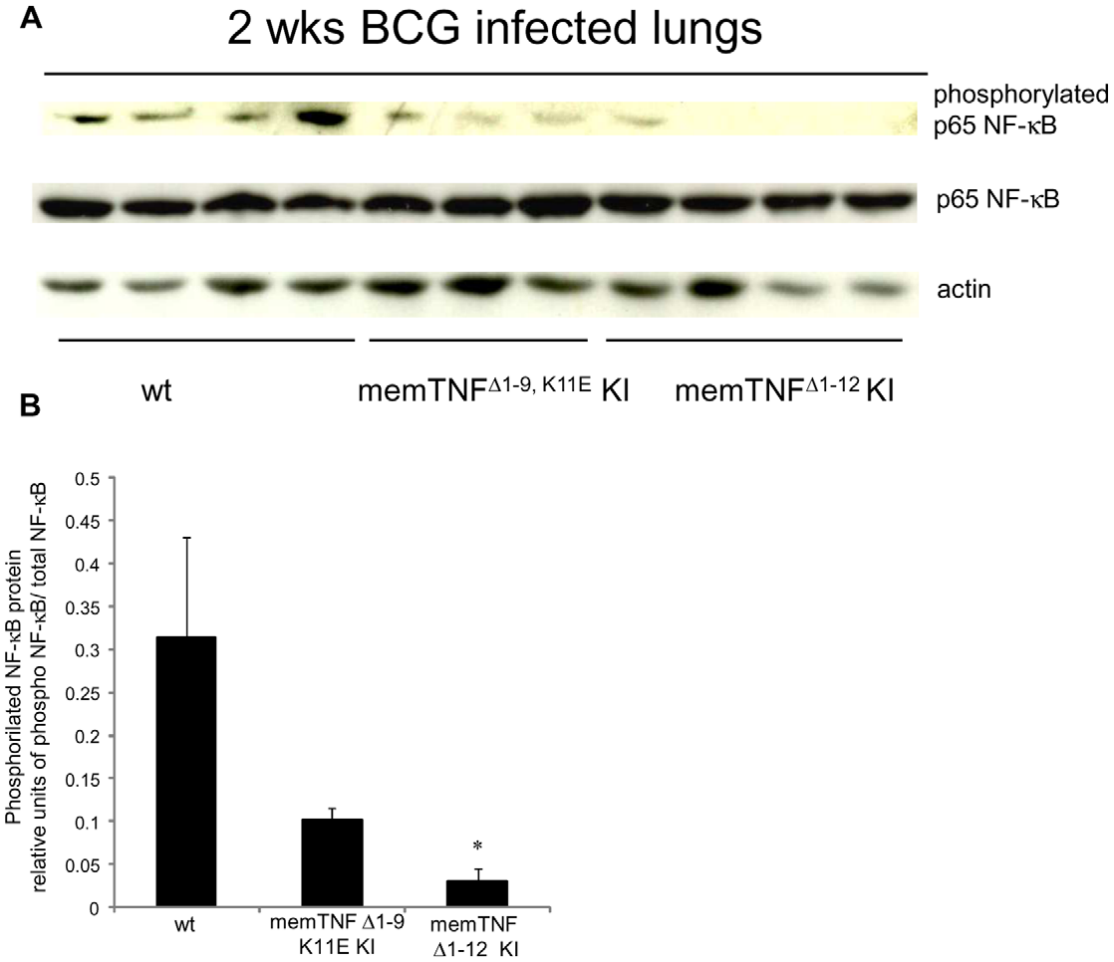
Decreased pulmonary phosphorylated p65 NF-κB in memTNF^Δ1–12^ KI mice after 2 weeks of *M. bovis* BCG infection. (A) Phosphorylated and unphosphorylated p65 NF-κB and actin protein expressions were detected in lungs by western blot 2 weeks after *M. bovis* BCG infection. (B) Quantification of phosphorylated and unphosphorylated p65 NF-κB on western blot was done by Quantity One® analysis software on two different experiments. Results are expressed as mean ± SEM of relative units of phosphorylated p65 NF-κB/total p65 NF-κB (*n* = 4–5 mice/group) (*, p<0.02).

Serum amounts of RANTES at 2 weeks infection were reduced in mutant mice, but at 4 weeks, levels were only lower in memTNF^Δ1–12^ KI and TNF−/− mice compared to wild-type mice (Fig. 3E). RANTES lung amounts were increased at 4 weeks of infection in memTNF^Δ1–9,K11E^ KI and in memTNF^Δ1–12^ KI mice compared to wild-type mice (Fig. 3F). MCP-1 serum levels were decreased in mutant mice at early infection as also observed for lung levels in all mutant mice, but at 4 weeks infection they were increased in memTNF^Δ1–12^ KI and TNF−/− mice but not in memTNF^Δ1–9,K11E^ KI mice compared to wild-type mice, suggesting an enhanced activity of lung macrophages (Fig. 3G, H). A general pattern of activation of inflammatory mediators revealed a delay in mutant mice at early infection which was attenuated in memTNF^Δ1–9,K11E^ KI mice. At late infection in mice suffering of progressive disease, an exacerbation of cytokine release was observed such as in memTNF^Δ1–12^ KI and TNF−/− mice whereas memTNF^Δ1–9,K11E^ KI mice exhibited normal or attenuated responses. To gain insight into the deficient pattern of activation observed at two weeks infection in memTNF KI mice, NF-κB phosphorylation patterns were analyzed by western blot. The pulmonary protein expression of the phosphorylated form of the p65 NF-κB subunit was compared with the unphosphorylated form. Phosphorylated p65 NF-κB was reduced in memTNF^Δ1–9,K11E^ KI mice but significantly much lower in memTNF^Δ1–12^ KI after *M. bovis* BCG infection indicating impaired cell activation in memTNF^Δ1–12^ KI mice (Fig. 4).

To explore whether the deficient pattern of cytokines in the circulation observed in mutant mice corresponds to the ability to mobilize and activate inflammatory cells to the site of infection, circulating activated inflammatory cells were characterized at early infection. Peripheral blood leucocytes after 1 week of *M. bovis* BCG infection were analyzed in memTNF^Δ1–9,K11E^ KI and memTNF^Δ1–12^ KI mice. Blood granulocytes (Gr1^+^) were lower in memTNF^Δ1–12^ KI mice which showed no difference before and after *M. bovis* BCG infection (Fig. S2A, B). In addition, the proportion of monocytes CD11b^+^ Gr1^−^ (resident macrophages) and CD11b^+^ Gr1^+^ (inflammatory macrophages) were also lower in memTNF^Δ1–12^ KI mice (Fig. S2C, D). These data suggest that the capacity to activate NF-κB and inflammatory cells, and to release cytokines and chemokines at early infection is impaired in memTNF^Δ1–12^ KI mice and this may be critical in the progression of the infection.

### Deficient IFN-γ antigen-specific production in memTNF^Δ1–12^ but not in memTNF^Δ1–9,K11E^ KI mice

**Figure 5:**
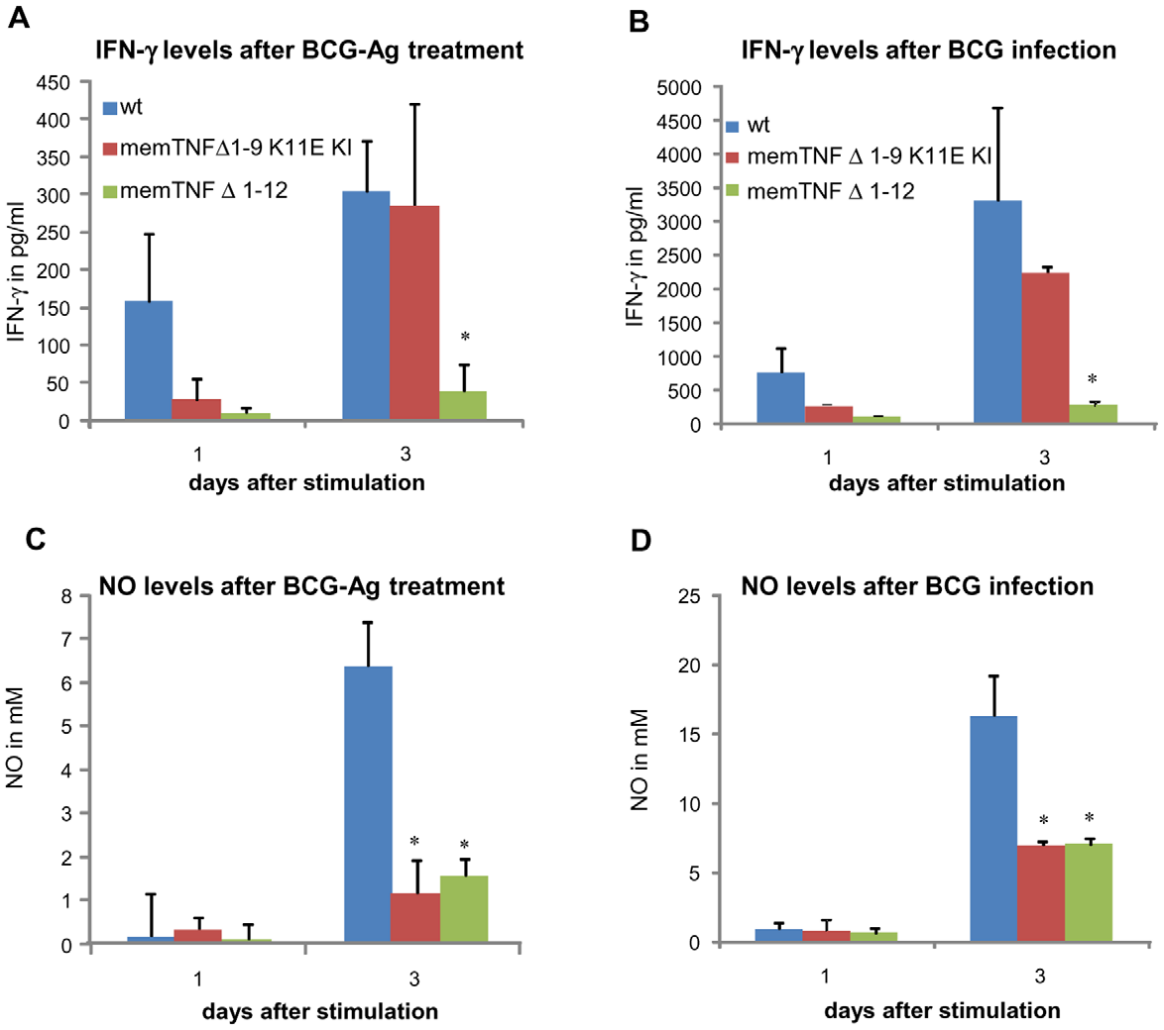
Antigen-specific IFN-γ and nitric oxide release from splenocytes of *M. bovis* BCG-infected mice. IFN-γ and NO (nitric oxide) were evaluated in culture supernatant from cells incubated with antigens derived from *M. bovis* BCG (A and C) or with 10^3^ viable *M. bovis* BCG (B and D). Values are represented as mean ± *SEM* (*n* = 3 animals per group, assayed in triplicate). * p<0.04.

**Figure 6:**
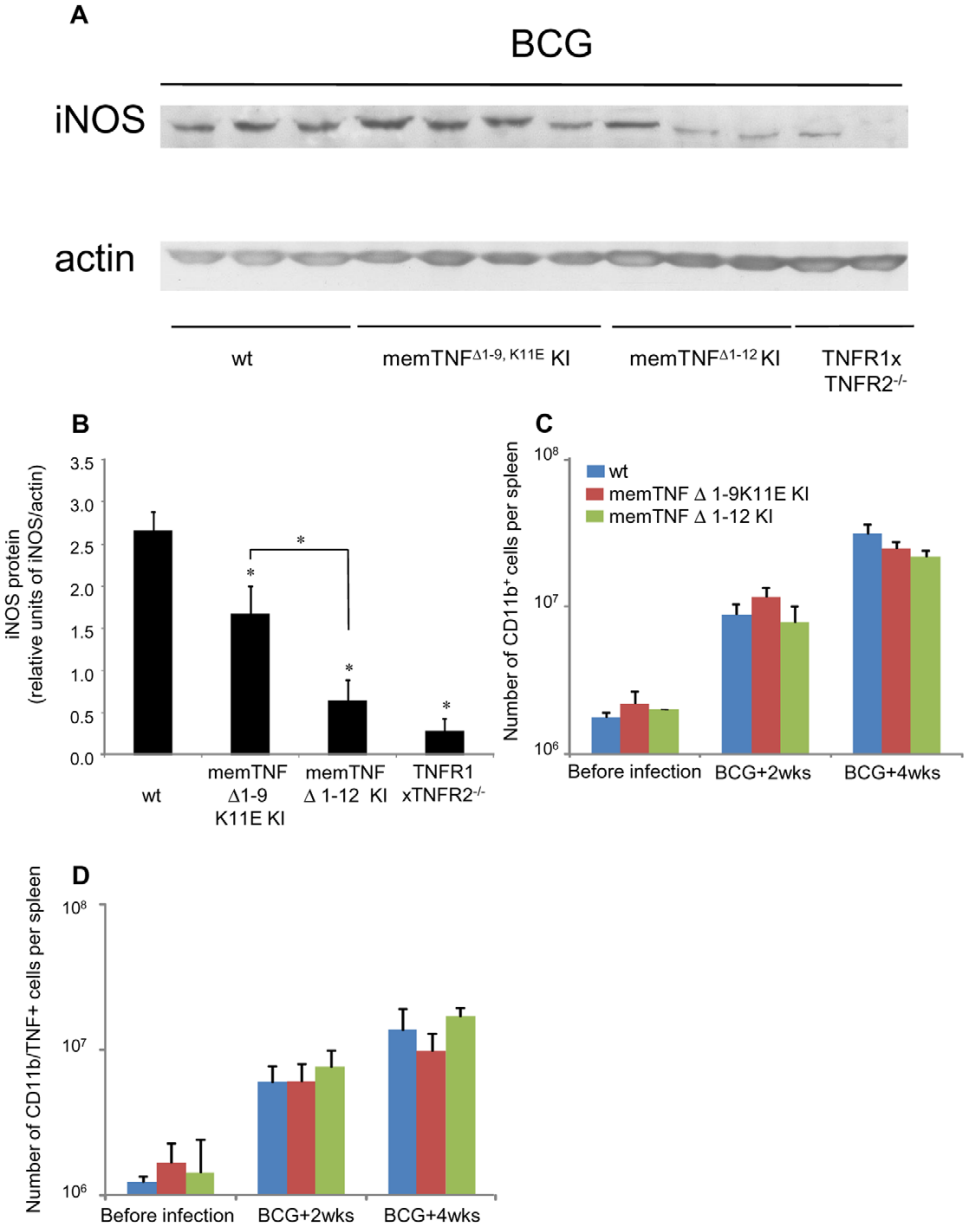
Decreased iNOS protein in memTNF KI but similar number of splenic CD11b^+^/TNF^+^ cells 4 weeks after *M. bovis* BCG infection. (A) Spleen iNOS protein expression was detected by western blot 4 weeks after *M. bovis* BCG infection. (B) iNOS protein was quantified by Image Quant software. Results are expressed as mean ± SEM of relative units of iNOS/actin (*n* = 4, except for TNFR1/TNFR2−/− mice *n* = 2 per group). *, p<0.04. FACS analyses showing the number of CD11b^+^ cells (C) and of CD11b^+^/TNF^+^ (D) in the spleen of uninfected, 2 and 4 weeks infected mice. Results are shown as mean ± SEM of positive cells per spleen (*n* = 4). *, p<0.01 compared to wild-type mice.

We further analyzed the capacity of lymphocytes to respond to antigen-specific stimulation to explore any difference between memTNF^Δ1–9,K11E^ KI and memTNF^Δ1–12^ KI mouse Th1 type immune responses. IFN-γ production was assessed after antigen-specific stimulation of splenocytes from 4 weeks *M. bovis* BCG-infected mice with BCG culture proteins or with living *M. bovis* BCG. MemTNF^Δ1–9,K11E^ KI splenocytes released comparable amounts of IFN-γ as wild-type, whereas memTNF^Δ1–12^ KI splenocytes dramatically lacked IFN-γ production (Fig. 5A) indicating a deficiency in T-antigen-specific cell activation in memTNF^Δ1–12^ but not in memTNF^Δ1–9,K11E^ KI mice. Splenocyte activation with live *M. bovis* BCG gave similar results; IFN-γ production was lower in memTNF^Δ1–12^ KI cells (Fig. 5B). We also assessed antigen-activated Nitrite/Nitrate as a surrogate of NO production, which is mainly released by macrophages, and observed that NO production was similar in memTNF^Δ1–9,K11E^ KI and memTNF^Δ1–12^ KI splenocytes. Amounts were much lower than in wild-type cells after antigen activation and also after *M. bovis* BCG infection, indicating the importance of soluble TNF for iNOS activity of macrophages (Fig. 5C, D). We further asked if IFN-γ antigen-specific deficient activation of memTNF^Δ1–12^ cells could be attributed to differences in splenocyte populations in infected mice. But surprisingly, we found that the number of CD4^+^ T cells was 4 times higher in memTNF^Δ1–12^ KI compared to memTNF^Δ1–9,K11E^ KI, and wild-type mice (Fig. S3A). However, the total number of CD11b^+^ was comparable in memTNF^Δ1–9,K11E^ KI, memTNF^Δ1–12^ KI and wild-type mice (Fig. 6C) suggesting that memTNF cannot replace soluble TNF in activating splenic iNOS *ex-vivo*.

### iNOS levels are reduced in *M. bovis* BCG-infected memTNF^Δ1–12^ KI mice compared to memTNF^Δ1–9,K11E^ KI mice

Activation of iNOS is a bactericidal mechanism essential for *M. bovis* BCG clearance and mouse survival [19]. The expression of iNOS protein in the spleen at 4 weeks post-infection was evaluated by western blot. MemTNF^Δ1–9,K11E^ KI, memTNF^Δ1–12^ KI and TNFR1/TNFR2−/− mice showed decreased iNOS protein expression compared to wild-type mice (Fig. 6A). In agreement with previous experiments, memTNF^Δ1–9,K11E^ KI mice had higher iNOS levels than memTNF^Δ1–12^ KI mice, suggesting that memTNF^Δ1–9,K11E^ activates iNOS *in vivo* more efficiently than memTNF^Δ1–12^. These observations were confirmed by the normalization of the iNOS band with actin (Fig. 6B). These results indicate that iNOS activation is deficient in memTNF^Δ1–12^ KI compared to memTNF^Δ1–9,K11E^ KI mice. We asked if the deficiency of memTNF^Δ1–12^ KI mice was due to reduced number of macrophages or to a differential expression of transmembrane TNF on macrophages and assessed the total number of CD11b^+^ and CD11b^+^/TNF^+^ cells in spleen before and after the infection (Fig. 6C, D). The number of macrophages expressing TNF was similar in wild-type, memTNF^Δ1–9,K11E^ KI and memTNF^Δ1–12^ KI mice indicating that this was not accounting for deficient iNOS expression. In contrast, the number of CD4^+^ T cells expressing TNF was higher in memTNF^Δ1–12^ KI than in memTNF^Δ1–9,K11E^ KI and wild-type mice (Fig. S3B). Indeed, the lack of iNOS expression in memTNF^Δ1–12^ KI mice was not due to reduced number of neither macrophages nor CD4 T cells expressing memTNF. These data indicate that interaction of memTNF with both soluble and membrane TNFRs can be implicated in protection and susceptibility to the infection.

### Alteration of TNF receptors in memTNF^Δ1–12^ KI mice upon *M. bovis* BCG infection

**Figure 7:**
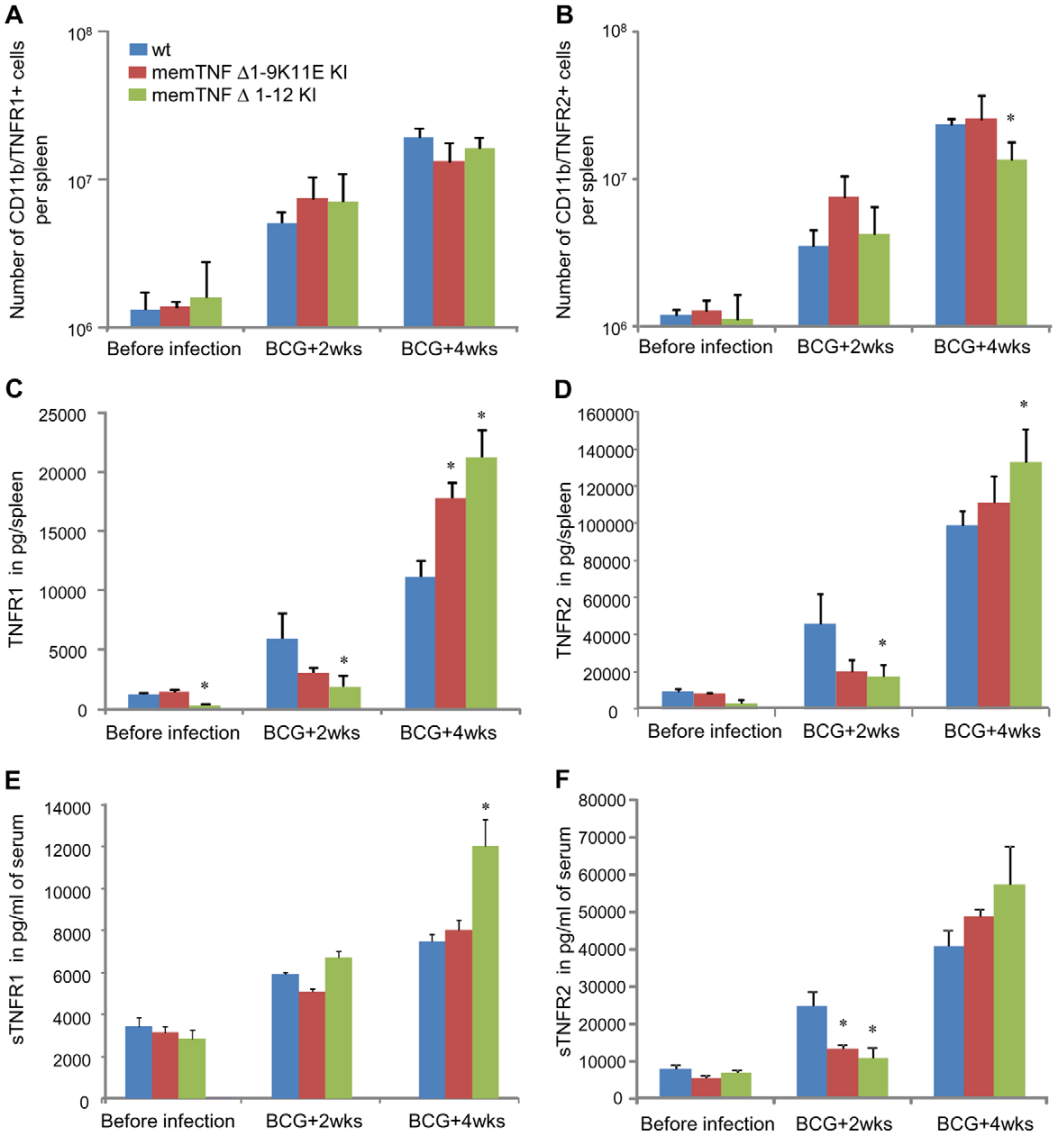
Lower number of macrophages expressing membrane bound TNFR2 but higher soluble TNFRs in memTNF^Δ1–12^ KI mice. FACS analyses showing number of spleen CD11b^+^ cells expressing membrane TNFR1 (A) or TNFR2 (B) before or during *M. bovis* BCG infection. Results are shown as means ± SEM of spleen cell numbers (*n* = 3–4). *, p<0.02 compared to wild-type mice. Total amounts of TNFR1 (C) and TNFR2 (D) were evaluated on total spleen extracts during *M. bovis* BCG infection. Results are shown as means ± SEM of pg per spleen (*n* = 4–6). *, p<0.05 compared to wild-type mice. Amounts of solTNFR1 (E) and solTNFR2 (F) were assessed in the serum of mice before and after infection. Results as represented as means ± SEM of pg/ml of serum (*n* = 6–10). *, p<0.05 compared to wild-type.

To investigate whether regulatory mechanisms of membrane TNF receptor as well as TNF receptor shedding could explain the enhanced sensitivity of memTNF^Δ1–12^ KI mice to intracellular bacterial infection, we analysed cells expressing TNFR1 and TNFR2 and total amounts of TNF receptors in mouse spleen before and after the infection. FACS analyses showed that the number of spleen macrophages expressing TNFR1 was similar in wild-type, memTNF^Δ1–9,K11E^ KI, and memTNF^Δ1–12^ KI mice. In contrast, after 4 weeks of infection, TNFR2^+^ macrophages were reduced in memTNF^Δ1–12^ KI compared to wild-type and memTNF^Δ1–9,K11E^ KI mice indicating a decreased signalling through TNFR2 which is considered to play an important role in memTNF activity (Fig. 7A, B). TNFR2 is cleaved at the cell surface by the TACE (ADAM 17) to form the soluble (s) TNFR2 that can antagonize the activity of solTNF and memTNF. Evaluation of the total amount of TNF receptors by ELISA in spleen homogenates revealed that TNFR1 and TNFR2 levels were lower at 2 weeks infection in memTNF^Δ1–12^ KI mice, but were significantly increased at 4 weeks infection when mice showed disease symptoms (Fig. 7C, D). It is of interest that the amounts of TNFR1 increased 100-folds after infection in memTNF^Δ1–12^ KI mouse spleen whereas in memTNF^Δ1–9,K11E^ KI and wild-type mice this increases was only 12 and 9-folds, respectively. Similarly, *M. bovis* BCG-induced TNFR2 augmented in the spleen of memTNF^Δ1–12^ KI mice 38-folds whereas in memTNF^Δ1–9,K11E^ KI and wild-type mice was only 13 and 10-folds, respectively. However, the levels of spleen TNFR2 were much higher (6- to 15-folds) than those of TNFR1 before and after infection (Fig. 7C, D). Similarly to the spleen, circulating sTNFR2 amounts were lower in memTNF KI mice and increased at 4 weeks infection suggesting that spleen pattern reflects sTNFR2. The amounts of sTNFR1 were significantly higher in memTNF^Δ1–12^ KI mice but those of sTNFR2 were again much higher than sTNFR1 indicating a major role in memTNF inhibition (Fig. 7E, F). These data suggest that TNFRs and in particular TNFR2 can be released more abundantly by activated macrophages during an infection leading to neutralization of memTNF^Δ1–12^ molecules which can be critical in severe progressive infection.

### Altered *M. bovis* BCG-induced nitric oxide, cytokine and chemokine responses and NF-κB activation of bone marrow-derived macrophages from memTNF^Δ1–12^ KI mice

**Figure 8:**
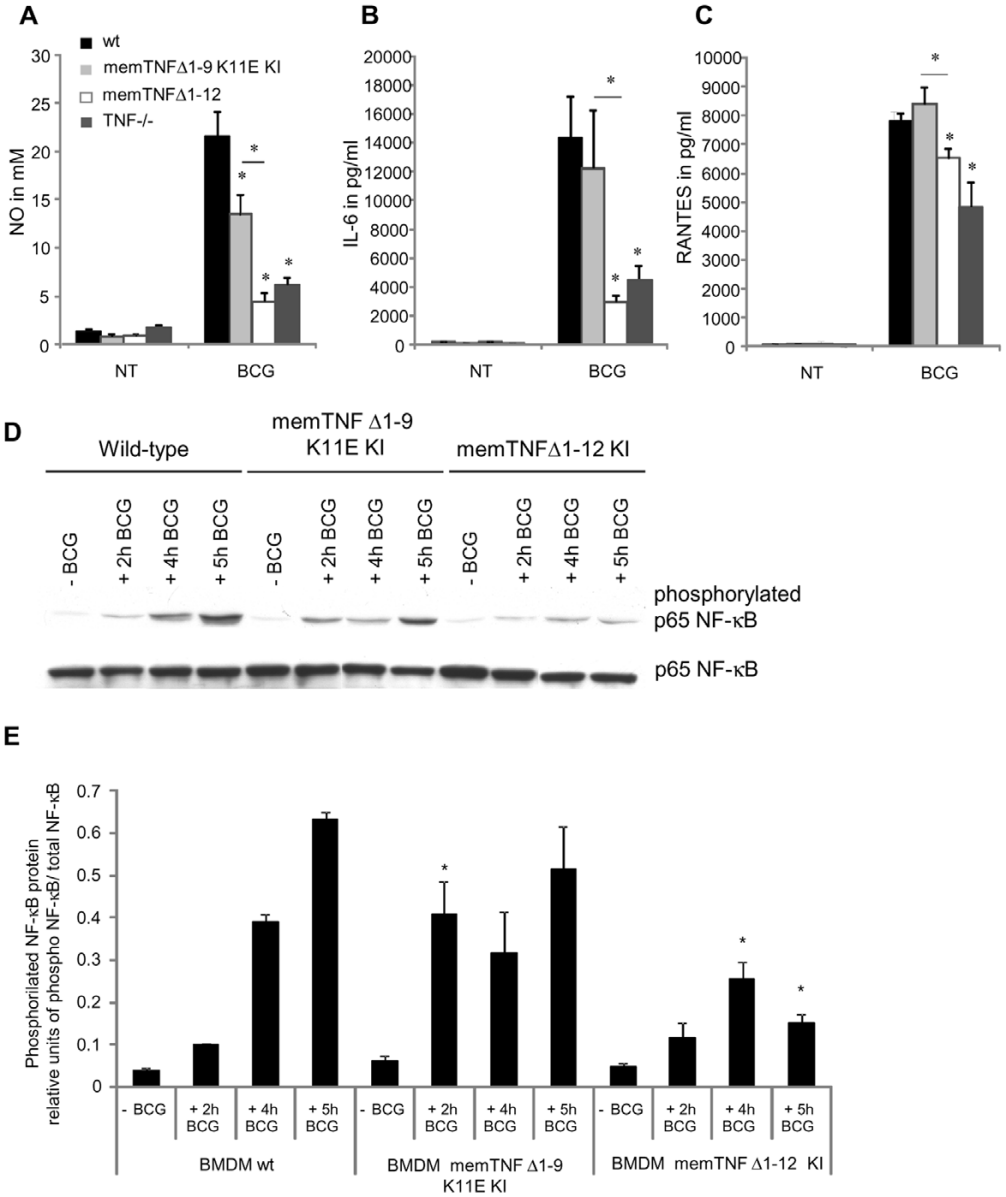
BMDM nitric oxide, IL-6, RANTES production and p65 NF-κB activation by *M. bovis* BCG infection. NO (A), IL-6 (B) and RANTES (C) levels were assessed in supernatant of BMDM 24 hours after *M. bovis* BCG infection. These results are representative of two independent experiments and values are represented as mean ± *SEM* (*n* = 3 animals per group, assayed in triplicate) *p<0.05. (D) Phosphorylated and unphosphorylated p65 NF-κB proteins were detected by western blot in BMDM not infected or infected with *M. bovis* BCG at different time points. (E) Quantification of phosphorylated p65 NF-κB on western blot was done by Quantity One® analysis software on two different experiments. Results are expressed as mean ± SEM of relative units of phosphorylated p65 NF-κB/total p65 NF-κB (*n* = 2/group). *, p<0.05 compared to wild-type BMDM.

To explore whether the differences observed between the two memTNF KI mice reflect a deficiency in macrophage activity of memTNF^Δ1–12^ KI mice, bone marrow-derived macrophages (BMDM) were infected with *M. bovis* BCG and production of NO, IL-6 and RANTES was assessed. Production of NO was significantly lower in memTNF^Δ1–12^ KI than in memTNF^Δ1–9,K11E^ KI BMDM although the latter was reduced when compared to wild-type BMDM (Fig. 8A). *M. bovis* BCG-induced IL-6 and RANTES was similar or increased in memTNF^Δ1–9,K11E^ KI and in wild-type BMDM but lower in memTNF^Δ1–12^ KI and TNF−/− BMDM (Fig. 8B, C).

To gain insight into difference in *M. bovis* BCG-induced cellular activation of the two memTNF molecules, NF-κB phosphorylation patterns were analyzed by western blot. Phosphorylated p65 NF-κB progressively increased in wild-type BMDM up to 5 hours after *M. bovis* BCG infection. In memTNF^Δ1–9,K11E^ KI BMDM, the activation of phosphorylated p65 NF-κB was faster than in wild-type BMDM and was already increased 2 hours after *M. bovis* BCG infection to similar levels as observed in wild-type BMDM 4 hours post-infection. In contrast, in *M. bovis* BCG-infected memTNF^Δ1–12^ KI BMDM, phosphorylation of p65 NF-κB protein was significantly reduced which confirms that memTNF^Δ1–12^ KI BMDM but not memTNF^Δ1–9,K11E^ KI BMDM are deficient in NF-κB activation (Fig. 8D, E).

## Discussion

The role of transmembrane TNF has been analyzed in different systems and it is now accepted that memTNF mediates important activities at the cellular level as well as in host defense mechanisms. Early reports of memTNF-mediated activities described the human memTNF^Δ1–12^ molecule which was shown to induce cell death and to be a predominant ligand for human TNFR2 [20], [21], [22]. The activities of mouse memTNF molecules used in the present study were originally analyzed by Decoster et al. on transfected mouse cells [23]. This study showed that deletion of the first 9 amino acids corresponding to mature TNF still led to production of soluble TNF, but that an additional Lys to Glu substitution at amino acid 11 gave rise to uncleavable membrane-bound TNF (memTNF^Δ1–9,K11E^) with biological activities similar to wild-type TNF. Comparison of memTNF^Δ1–9,^ ^K11E^ with the murine mutant memTNF^Δ1–12^ showed that the latter induced 3-fold lower GM-CSF responses and involved cooperative effects of TNFR1 and TNFR2 [23]. The present study explores these two memTNF molecules expressed in mouse lacking solTNF for their capacity to stimulate cell-mediated immune responses to intracellular bacteria. Our data indicate that memTNF^Δ1–9,K11E^ KI mice are substantially more resistant to *M. bovis* BCG infection than memTNF^Δ1–12^ KI mice, as demonstrated both by lower survival and significantly higher bacterial load, severity of pulmonary lesions and deficient pulmonary NF-κB activation in memTNF^Δ1–12^ KI mice. The deficiency of memTNF^Δ1–12^ KI mice to *M. bovis* BCG was confirmed by patterns of cytokine/chemokine and inflammatory cells that resembled TNF−/− whereas memTNF^Δ1–9,K11E^ KI mice were more similar to wild-type mice or showed an intermediate phenotype that was in general sufficient to mount an efficient immune response to *M. bovis* BCG infection.

These two memTNF mouse strains have been previously infected with *M. bovis* BCG using two different routes and dosages. All memTNF^Δ1–9,K11E^ KI mice survived to an intranasal infection with 10^6^ CFU and only 50% of memTNF^Δ1–12^ KI mice survived to an intravenous infection (2×10^6^ ) but infection conditions cannot be properly compared [17], [18] (see Table 1). Infection with *L. monocytogenes* showed difference in protection of the two memTNF strains as 100% memTNF^Δ1–9,K11E^ KI resisted [8] whereas only 10% memTNF^Δ1–12^ KI mice survived to 10^4^ CFU [5]. Similarly, infection with *M. tuberculosis* showed different susceptibilities since memTNF^Δ1–9,K11E^ KI mice infected with 50–100 CFU died 150–230 days whilst memTNF^Δ1–12^ KI mice infected with 10–30 CFU died 50–170 days after (Table 1). *M. bovis* BCG-infected mice expressing memTNF^Δ1–12^ but neither TNFR2 nor TNFR1 showed no significant differences in survival at day 50 compared to memTNF^Δ1–12^ KI mice although memTNF^Δ1–12^ KI-TNFR2−/− started to succumb earlier. A previous report has shown that 45 days after *M. tuberculosis* infection about 20% of memTNF^Δ1–12^ KI-TNFR2−/− died while about 50% of memTNF^Δ1–12^ KI-TNFR1−/− and memTNF^Δ1–12^ KI mice succumbed [17]. Similarly to results shown for *M. tuberculosis* infection, the pattern of pulmonary bacterial load was not significantly different in memTNF^Δ1–12^ KI compared to memTNF^Δ1–12^ KI-TNFR2−/− and memTNF^Δ1–12^ KI-TNFR1−/− mice after *M. bovis* BCG infection. Our interpretation is that memTNF-TNFR2 interaction may play a role at early infection but then, the incidence on bacterial load at 4 weeks infection is minimal as protection can be mediated by cooperation of both TNF receptors, and in addition, other regulatory mechanisms such as TNFR regulation can be critical in disease outcome.

To further explore other factors of interest favouring protection, we analysed the capacity of spleen cells to respond to antigens and to produce IFN-γ and observed that memTNF^Δ1–12^ KI were deficient whereas memTNF^Δ1–9,K11E^ KI cells were similar to wild-type cells. Surprisingly, the number of CD4^+^ T cells was four-fold higher in the spleen of memTNF^Δ1–12^ KI indicating that memTNF^Δ1–12^ KI cells are unable to respond to antigenic stimulation spite the moderated expansion of spleen CD4^+^ T cells. Previous report identified TNF as a negative regulator of Th1 type immune response during intracellular bacterial infection and showed an uncontrolled expansion of spleen CD4^+^ T cells, a marked increase of circulating and antigen-specific IFN-γ in TNF−/− mice [24]. Our data suggest that memTNF molecule appears to restrain exacerbation of Th1 immune response caused by lack of TNF. This is of interest considering the possibility of using the novel class of TNF inhibitors, dominant-negative (DN)-TNF biologics, that antagonize solTNF but not memTNF. We have shown that DN-TNF biologics protects from hepatitis but did not alter immunity against mycobacterial infections, presumably by maintenance of physiological memTNF signalling [7], [25].

We asked if anti-mycobacterial effector mechanisms *in vivo* such as iNOS were impaired and found that iNOS was much lower in the spleen of memTNF^Δ1–12^ KI than in memTNF^Δ1–9,K11E^ KI mice, confirming a decrease in bactericidal mechanisms of memTNF^Δ1–12^ form. To further explore the mechanisms that can influence the activity of memTNF, we focus on the investigation of spleen macrophages expressing TNF and TNFRs during the infection. The number of macrophages expressing TNF increased with the infection similarly in the three groups of mice. Interestingly, memTNF^Δ1–12^ KI mice showed a decrease in the number of macrophages expressing TNFR2 suggesting that macrophages cannot respond to *M. bovis* BCG-activated memTNF^Δ1–12^. In addition, the observation that total amount of TNFR2 is higher in spleen homogenates from memTNF^Δ1–12^ KI mice indicates over-shedding of TNFR2 and that a regulatory mechanism of memTNF inhibition may be involved in the pathogenicity. We have previously shown that *M. bovis* BCG infection induces high levels of serum TNF in parallel to the release of TNF receptors and this is influenced by iNOS activity since iNOS−/− mice showed enhanced serum levels of TNF and TNFR2 [19]. We have also reported that circulating *M. bovis* BCG-induced TNF is not bioactive but rapidly neutralized by sTNFRs during an infection [25]. The presence of high amounts of sTNFR2 as observed in iNOS−/− mice indicates abnormal TACE activity in memTNF^Δ1–12^ KI cells leading to memTNF inactivation by sTNFR2. Although that both receptors are enzymatically digested from cells, the amounts of sTNFR2 are much higher (in general 5 to 15-folds) than sTNFR1 which means that the neutralizing activity of sTNFR2 may play a predominant role compared to sTNFR1. Several studies have pointed out the critical role of sTNFR2 in bacterial and viral infections. A report showed that apoptosis of *M. tuberculosis*-infected alveolar macrophages was inhibited by the release of TNFR2 that inactivated TNF [26]. It has been shown that the immunopathology associated with T-cell-mediated influenza clearance was abrogated in TNFR2−/− mice and that TNFR2 was not need for memTNF to activate MCP-1 activation, whereas both receptors were required for MCP-1 expression by solTNF in alveolar epithelial cells showing the complexity of sol/memTNF-mediated activities with receptors in different systems [27].

SolTNF can mediate optimal protection against mycobacterial infections by providing exocrine and paracrine signalling to many cell types. In contrast, the signal of transmembrane TNF is restricted to cell-to-cell contact which limits the intensity of the activity. Our study shows that both memTNF molecules mediate protection but the effect of memTNF^Δ1–9,K11E^ appears clearly higher than the memTNF^Δ1–12^ molecule. The fact that memTNF^Δ1–9,K11E^ KI mice can eventually become sensitive and succumb to the infection highlights a complex regulation of factors mediating innate and adaptive immune responses that can be subjected to individual situations. Among these factors the equilibrium between memTNF and cell-bound and sTNFRs can play a critical role in host defense against intracellular bacteria *in vivo*. Our hypothesis is that the activities of memTNF can be modulated by the levels of membrane and soluble TNF receptors which may influence the outcome of the infection thus explaining why memTNF KI mice can be protected or can die from the infection. We have previously shown, using transgenic mice expressing human soluble TNFR1-IgG, that expression levels of the TNF neutralizing transgene determined the enhanced protection to *M. bovis* BCG infection or the higher susceptibility of mice compared to littermate control mice. We found that low levels of soluble receptors were activating TNF expression and macrophage effector mechanisms whilst high levels of soluble receptors were neutralizing both sol and memTNF blocking all protective TNF-mediated activities [16]. The immune cellular activation was attributed to reverse signaling of memTNF binding to solTNFRs. We hypothesize that a precise regulation of memTNF activity can be orchestrated by membrane and soluble TNFRs and an unbalance of this regulatory and complex system can modify the outcome of the disease.

We finally investigate if memTNF bone-marrow derived macrophages could show different responses upon *M. bovis* BCG infection *in vitro* and observed that macrophages from memTNF^Δ1–12^ KI mice produced lower amounts of cytokines/chemokines and showed an impaired p65 NF-κB phosphorylation pattern indicating that the deficiency is already on memTNF^Δ1–12^ KI macrophages which will influence the host immune response. These data suggest that the small difference of the two memTNF molecules results in major effects in signalling and memTNF-mediated activities.

**Figure 9:**
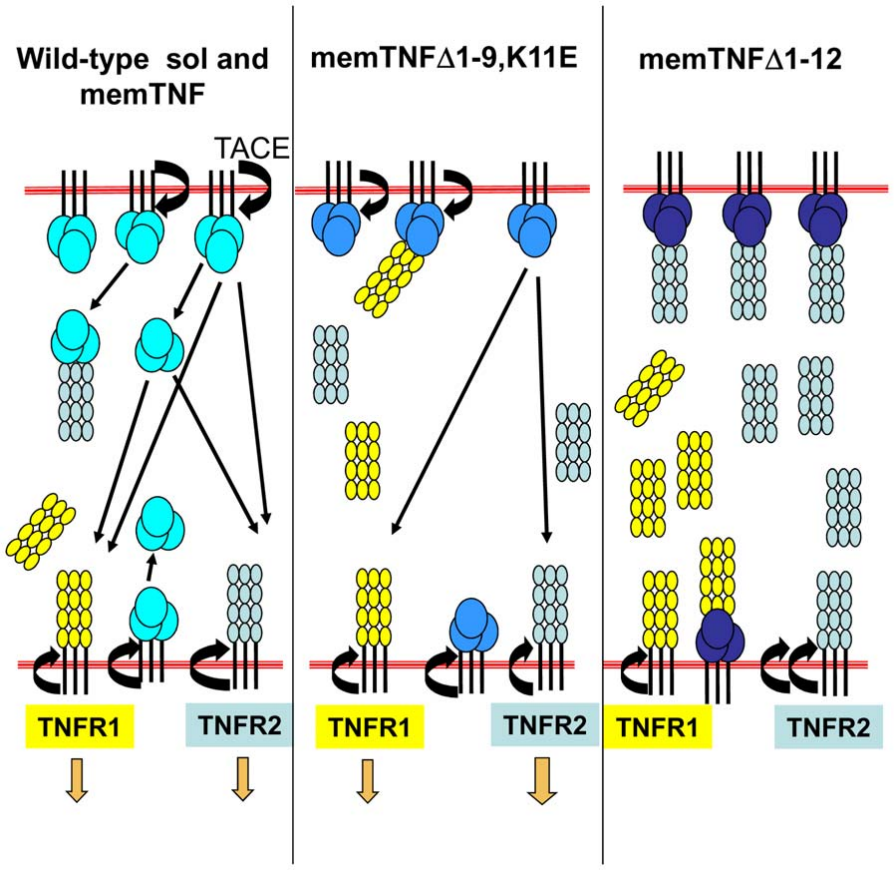
Hypothesis explaining differences between memTNF molecules. This figure presents the three situations explored in this work: wild-type TNF molecules which are digested by the TACE, and the two memTNF forms which cannot be digested and exert their activities by cell-to-cell contact. The difference between memTNF^Δ1–9,K11E^ and memTNF^Δ1–12^ molecules may reside in the possibility of the TACE to bind but not cleave the memTNF ^Δ1–9,K11E^, but in memTNF^Δ1–12^ the TACE binding site is not accessible and the protease activity is exerted on TNFR2 which became more frequently soluble thus neutralising memTNF^Δ1–12^ activities.

The mechanisms explaining the differences between memTNF molecules with only a minor change of 3 amino acids at positions 10–12 are still not elucidated. Some hypothesis can be envisaged to understand why such important consequence can occur *in vivo*. We hypothesize that TACE can still bind to memTNF^Δ1–9,K11E^ although unable to digest whereas the deletion of 3 amino acids prevent the binding of TACE to memTNF^Δ1–12^. Upon TNF activation, TACE activity is directed to TNFR2 which can be found decreased on macrophages but substantially increased as solTNFR2 form that will neutralize memTNF thus preventing its biological activities (Figure 9).

## Materials and Methods

### Mice

C57BL/6 mice, memTNF^Δ1–9,K11E^ KI mice [6], memTNF^Δ1–12^ KI mice [5] memTNF^Δ1–12^×TNFR1−/− mice, memTNF^Δ1–12^×TNFR2−/− mice, TNFR1×TNFR2−/− mice and TNF−/− [28] mice were maintained under conventional conditions in the animal facility of the Medical Faculty, University of Geneva. All animal experiments were carried out in accordance with institutional guidelines and were approved by the academic ethical committee on animal experimentation and the Cantonal Veterinary Office from Geneva, authorization number: 31.1.1005/3202/2.

### *M. bovis* BCG infection

Mice were infected intravenously (i.v.) with 10^7^ living *M. bovis* BCG Connaught [29], or BCG Pasteur GFP [30] (used for survival curve in figure S1). Body weights were monitored during infection, and mice were sacrificed at 2 and 4 weeks post-infection, or 24 weeks post-infection for survival monitoring.

### Determination of colony forming units (CFU) from infected organs

The number of viable bacteria recovered from frozen organs was evaluated as previously described [19].

### Histological analyses

Histological analyses of liver were performed at 4 weeks after BCG infection. Livers were fixed in 4% buffered formaldehyde and embedded in paraffin for subsequent hematoxylin/eosin (H&E) staining. Evaluation of the lesions was done with Metamorph sofware.

### Evaluation of serum, organ and cell culture supernatant levels of cytokines and chemokines

For cytokine and chemokine detection, organs were homogenized in 0.04% Tween 80/saline buffer (125 mg of tissue/mL) as previously described [31]. IFN-γ, IL-12p40, IL-6, MCP-1 RANTES, sTNFR1 and sTNFR2 amounts were evaluated in serum, organ extracts or cellular supernatant by ELISA with a sensitivity of 5–2000 pg/ml.

### Western blot analysis

Splenic proteins were prepared and western blot performed as previously described [32]. Primary antibody was a polyclonal rabbit anti-mouse iNOS (Calbiochem, San Diego, CA; 1∶2000 dilution). A rabbit polyclonal anti-actin was used as control antibody. Goat anti-rabbit HRP was the secondary antibody (Bio-Rad, Hercules, CA; 1∶5000 dilution). Western blot was also used to measure expression of the phosphorylated form of NF-κB in lungs and bone marrow derived macrophages, which were lysed in RIPA buffer complete with phosphatase (phosSTOP, Roche, Germany) and protease inhibitors (Complete mini, Roche, Germany). Primary antibody was either a rabbit polyclonal anti-NF-κB p65 antibody (Santa Cruz Biotechnology, USA; 1∶500 dilution) or a rabbit monoclonal anti-phosphorylated NF-κB p65 antibody (Cell Signaling Technology, USA; 1∶1000 dilution). Goat anti-rabbit HRP was used as secondary antibody (Bio-Rad, USA; 1∶5000 dilution). Blots were developed with Immobilon western chemiluminescent HRP substrate kit (Millipore, USA). The density of bands was quantified by using Image Quant 3.3 measurement software (Molecular Dynamics, USA) or by Quantity One® analysis software (Bio-Rad, USA).

### Antigen-specific release of nitric oxide and cytokines from spleen cells

Mice were infected and sacrificed 4 weeks later, and spleen cells were prepared as previously described [15]. Briefly, spleen cells were treated for 5 min with a 0.155 M ammonium chloride/0.010 M potassium bicarbonate solution for erythrocyte lysis, washed and resuspended in DMEM+10% FCS. Cells were plated at 5×10^5^ cells per well in 96-well plates and were stimulated with either medium alone, living *M. bovis* BCG (10^3^ CFU/well), or *M. bovis* BCG culture protein extracts (17 mg/ml) for one or three days. Cell culture medium was harvested for NO and cytokine determination. Nitrite accumulation was evaluated in culture medium as an indicator of NO production by Griess reagent (1% sulfanilamide and 0.1% naphtylethylenediamide in 2.5% phosphoric acid). Absorption was measured at 550 nm and nitrite concentrations were determined by comparison with OD of the NaNO2 standards. Cytokines were determined in cell supernatants as described below.

### Flow cytometry analyses

Flow cytometry was performed on peripheral blood leukocytes and splenocytes. To obtain peripheral blood leukocytes, mice were bled one week after BCG infection, and flow cytometry was performed as previously described [33]. Spleen cells were obtained from uninfected and at 2 and 4 weeks after *M. bovis* BCG infection. Flow cytometry was performed using three- or four-color staining and analyzed with a FACSCalibur (BD Biosciences, Mountain View, CA). The following antibodies were used: anti-Gr1 (RB6-8C5) (eBioscience), anti-CD11b (clone M1/70) (BD Biosciences), anti-CD4 (clone GK1.5) (ImmunoTools GmbH, Germany), polyclonal anti-TNF, polyclonal anti-TNFR1 and polyclonal anti-TNFR2 (Hycult biotech). Staining was performed in the presence of a saturating concentration of 2.4G2 anti-Fcγ RII/III mAb. For spleen, data were calculated in total number of positive cells.

### Bone marrow-derived macrophage (BMDM) culture and treatment

Macrophages were derived from bone marrow cells as previously described [34]. Briefly, bone marrow cells were flushed aseptically from the femurs of mice and cultured in DMEM supplemented with 10% fetal calf serum and 20% L929 cell-conditioned medium [34]. After 6 days of culture, BMDM were infected with *M. bovis* BCG (60 000 CFU/well) during 2, 4, 5 or 24 hours. The supernatant was harvested for NO and cytokine determination.

### Statistical analyses

The one-way ANOVA was used for all analyses. *P* values<0.05 were considered as statistically significant.

## Supporting Information

Figure S1 **Survival curve and body weight of** ***M. bovis*** **BCG infected mice.** (A) Long-term survival of mice infected with living *M. bovis* BCG Pasteur (10^7^). (B) Body weight change after *M. bovis* BCG infection (*n* = 4–5 mice per group). Data from one representative experiment are shown. (C and D) CFU at 4 weeks after infection with 10^7^ CFU of *M. bovis* BCG Connaught were determined in lungs (C) and liver (D). Data are represented as individual values and horizontal bars indicate mean (*n* = 4–6 mice per group). Asterisks indicate statistically significant differences between wild type and indicated group (*, p<0.03).
(TIF)

Figure S2 **Peripheral blood mononuclear cells (PBMC) were reduced in memTNF^Δ1–12^ KI after 1 week of** ***M. bovis*** **BCG infection.** (A) PBMC before infection and 1 week after *M. bovis* BCG infection were stained with anti-Gr1 monoclonal antibody (mAb). PBMC were gated for cells with higher granularity (high side–scatter properties) to distinguish polymorphonuclear cells. Numbers indicate the mean percentages of Gr1^+^ polymorphonuclear cells. (B) Percentages of Gr1^+^ cells are shown (*n* = 3 mice per group) (*, p<0.04). (C) PBMC from the same group of mice before and 1 week after *M. bovis* BCG infection were stained with a combination of anti-CD11b and anti-Gr1 monoclonal antibodies (mAb) and were gated for cells with lower granularity (low side–scatter properties) to distinguish them from polymorphonuclear cells. (D) Percentage of cells is shown in histogram (*n* = 3 mice per group) (*, p<0.03).
(TIF)

Figure S3 **Decreased number of splenic CD4^+^ and CD4^+^/TNF^+^ cells 4 weeks after** ***M. bovis*** **BCG infection.** Number of CD4^+^ T (A) and CD4^+^/TNF^+^ T (B) cells in spleen at 4 weeks of *M. bovis* BCG infection were increased in memTNF^Δ1–12^ KI mice. Data were represented as means ± SEM of positive cell number per spleen (*n* = 3–4 mice per group) (*, p<0.02).
(TIF)
