# Supplementary figures and images for: Membrane-Bound TNF Induces Protective Immune Responses to M. bovis BCG Infection: Regulation of memTNF and TNF Receptors Comparing Two memTNF Molecules
Source: PLoS One. 2012 May 30;7(5):e31469. doi: 10.1371/journal.pone.0031469 (PMC3364241; doi:10.1371/journal.pone.0031469)

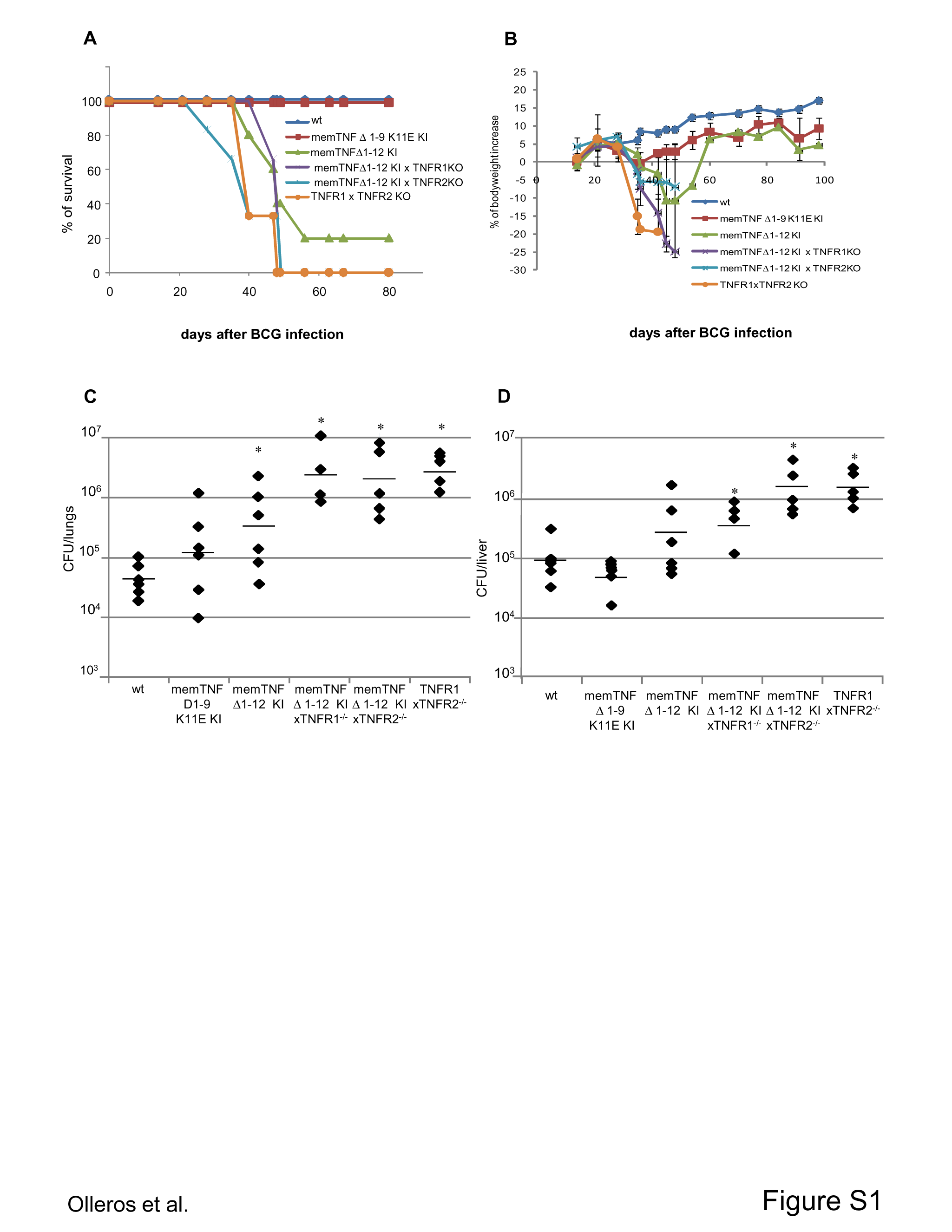

Supplement: Figure S1 — Survival curve and body weight of M. bovis BCG infected mice. (A) Long-term survival of mice infected with living M. bovis BCG Pasteur (107). (B) Body weight change after M. bovis BCG infection (n = 4–5 mice per group). Data from one representative experiment are shown. (C and D) CFU at 4 weeks after infection with 107 CFU of M. bovis BCG Connaught were determined in lungs (C) and liver (D). Data are represented as individual values and horizontal bars indicate mean (n = 4–6 mice per group). Asterisks indicate statistically significant differences between wild type and indicated group (*, p<0.03). (TIF) [file pone.0031469.s001.tif]

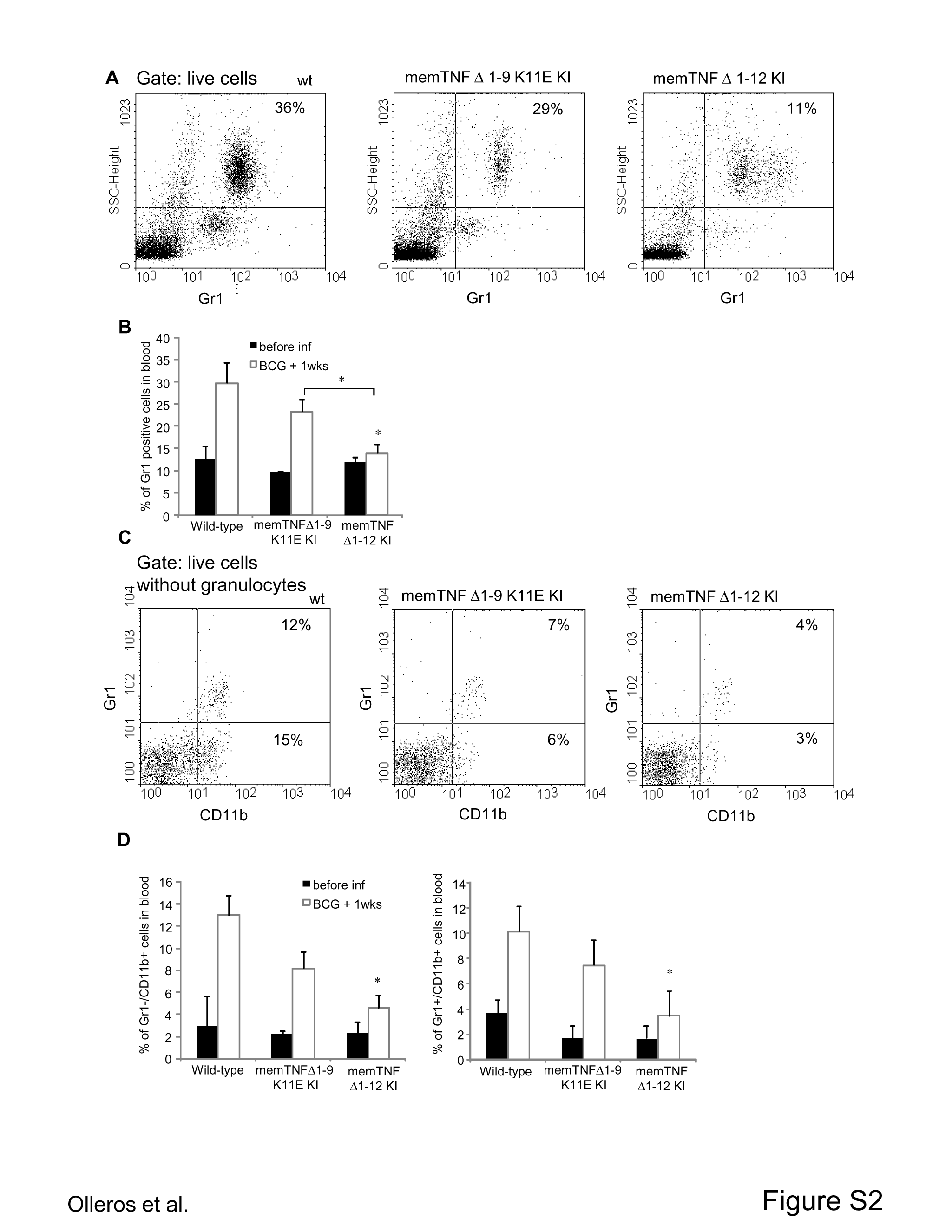

Supplement: Figure S2 — Peripheral blood mononuclear cells (PBMC) were reduced in memTNFΔ1–12 KI after 1 week of M. bovis BCG infection. (A) PBMC before infection and 1 week after M. bovis BCG infection were stained with anti-Gr1 monoclonal antibody (mAb). PBMC were gated for cells with higher granularity (high side–scatter properties) to distinguish polymorphonuclear cells. Numbers indicate the mean percentages of Gr1+ polymorphonuclear cells. (B) Percentages of Gr1+ cells are shown (n = 3 mice per group) (*, p<0.04). (C) PBMC from the same group of mice before and 1 week after M. bovis BCG infection were stained with a combination of anti-CD11b and anti-Gr1 monoclonal antibodies (mAb) and were gated for cells with lower granularity (low side–scatter properties) to distinguish them from polymorphonuclear cells. (D) Percentage of cells is shown in histogram (n = 3 mice per group) (*, p<0.03). (TIF) [file pone.0031469.s002.tif]

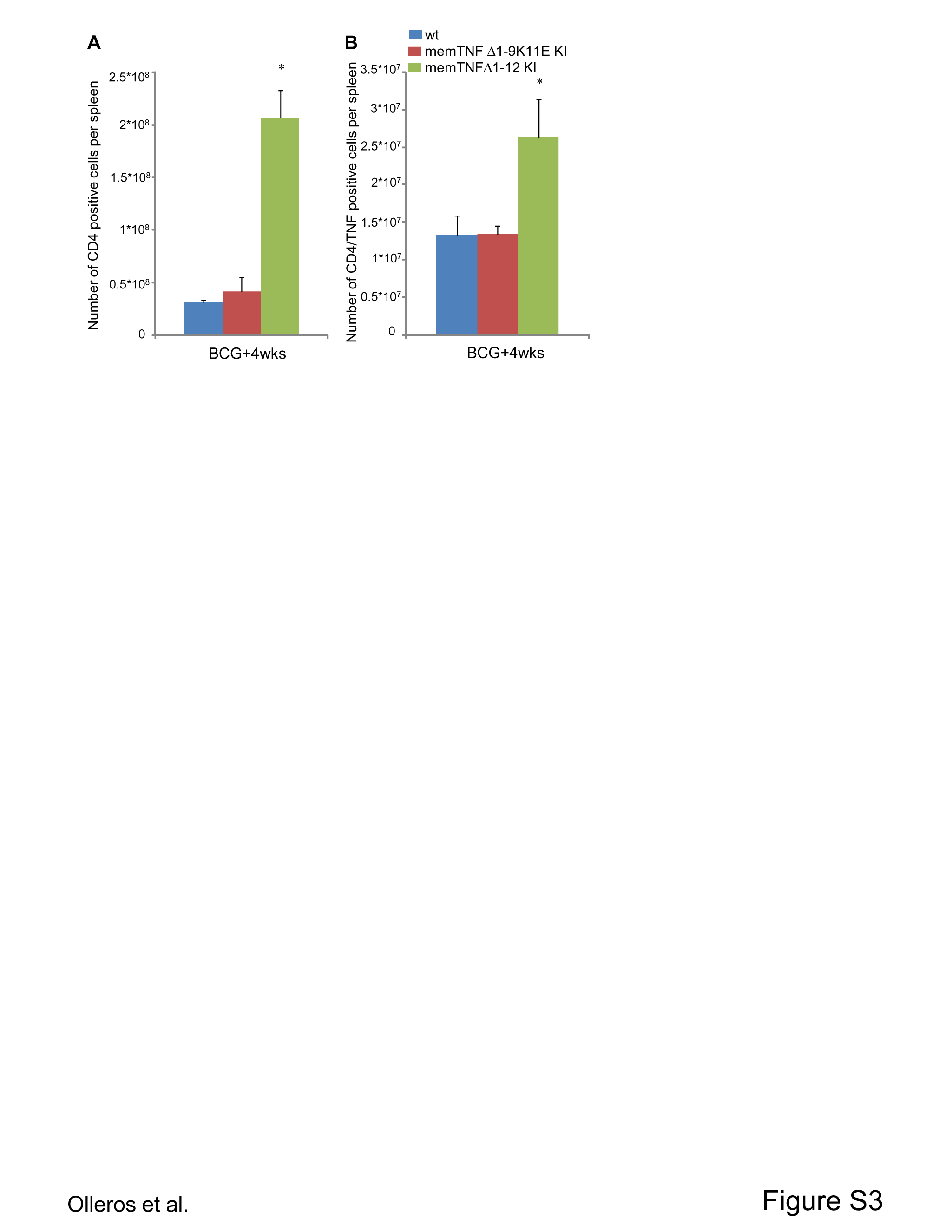

Supplement: Figure S3 — Decreased number of splenic CD4+ and CD4+/TNF+ cells 4 weeks after M. bovis BCG infection. Number of CD4+ T (A) and CD4+/TNF+ T (B) cells in spleen at 4 weeks of M. bovis BCG infection were increased in memTNFΔ1–12 KI mice. Data were represented as means ± SEM of positive cell number per spleen (n = 3–4 mice per group) (*, p<0.02). (TIF) [file pone.0031469.s003.tif]
